# Adiponectin Inhibits NLRP3 Inflammasome Activation in Nonalcoholic Steatohepatitis via AMPK-JNK/ErK1/2-NFκB/ROS Signaling Pathways

**DOI:** 10.3389/fmed.2020.546445

**Published:** 2020-11-05

**Authors:** Zhixia Dong, Qian Zhuang, Xin Ye, Min Ning, Shan Wu, Lungen Lu, Xinjian Wan

**Affiliations:** ^1^Digestive Endoscopic Center, Shanghai JiaoTong University Affiliated Sixth People's Hospital, Shanghai, China; ^2^Department of Gastroenterology, Shanghai General Hospital, Shanghai Jiao Tong University School of Medicine, Shanghai, China

**Keywords:** adiponectin, hepatocytes, NAFLD, NLRP3 inflamamasome, AMPK

## Abstract

Adiponectin, an adipose-derived adipokine, possesses a hepatoprotective role in various liver disorders. It has been reported that hypoadiponectinemia can affect with the progression of non-alcoholic fatty liver diseases (NAFLD). Inflammasome activation has been recognized to play a major role during the progression of NAFLD. This research aimed to explore the effect of adiponectin on palmitate (PA)-mediated NLRP3 inflammasome activation and its potential molecular mechanisms. Male adiponectin-knockout (adiponectin-KO) mice and C57BL/6 (wild-type) mice were fed a high-fat-diet (HFD) for 12 weeks as an *in vivo* model of non-alcoholic steatohepatitis (NASH). Serum biochemical markers, liver histology and inflammasome-related gene and protein expression were determined. In addition, the hepatocytes isolated from wide type mice were exposed to PA in the absence or presence of adiponectin and/or AMPK inhibitor. The activation of NLRP3 inflammasome was assessed by mRNA and protein expression. Furthermore, ROS production and related signaling pathways were also evaluated. In the *in vivo* experiments, excessive hepatic steatosis with increased NLRP3 inflammasome and its complex expression were found in adiponectin-KO mice compared to wild-type mice. Moreover, the expression levels of NLRP3 inflammasome pathway molecules (NFκB and ROS) were upregulated, while the phosphorylation levels of AMPK, JNK, and Erk1/2 were downregulated in adiponectin-KO mice compared with wild-type mice. In the *in vitro* study, PA increased lipid droplet deposition, NF-kB signaling and ROS production. Additionally, PA significantly promoted NLRP3 inflammasome activation and complex gene and protein expression in hepatocytes. Adiponectin could abolish PA-mediated inflammasome activation and decrease ROS production, which was reversed by AMPK inhibitor (compound C). Furthermore, the results showed that the inhibitory effect of adiponectin on PA-mediated inflammasome activation was regulated by AMPK-JNK/ErK1/2-NFκB/ROS signaling pathway. Adiponectin inhibited PA-mediated NLRP3 inflammasome activation in hepatocytes. Adiponectin analogs or AMPK agonists could serve as a potential novel agent for preventing or delaying the progression of NASH and NAFLD.

## Introduction

Non-alcoholic fatty liver diseases (NAFLD) contains a broad histopathological spectrum ranging from steatosis alone to non-alcoholic steatohepatitis (NASH), which can result in hepatic cirrhosis and even liver cancer (1, 2). NASH is highly associated with obesity, insulin resistance, hypertension, and diabetes mellitus (3). In recent years, with the changes in human diet and lifestyle, the incidence rates of NAFLD and NASH are increased at an alarming rate, which has become the leading cause of chronic hepatic diseases (4, 5). At present, there is no effective therapeutic drug for NAFLD treatment. Thus, it is of great significance to understand the pathogenesis of NAFLD. The “multiple-hit” hypothesis on NASH development has been widely spread and well-recognized (6). Recently, new insights into the molecular mechanism of NASH have been provided. Numerous studies have suggested the involvement of inflammasome activation in the pathogenesis of NASH (7–10).

Inflammasomes are large cytoplasmic multi-protein complexes that response to both exogenous and endogenous alerting signals through intracellular NOD-like receptor (NLR) (11, 12). NLR family pyrin domain-containing 3 (NLRP3) inflammasome, composing of NLRP3, the adaptor protein ASC and the effector molecule pro-caspase1, is well-characterized (13, 14). The activation of NLRP3 inflammasome usually requires two signals (15): the prime signal that promotes the transcription of inflammasome components through NFκB signaling activation (16), and the second signal is initiated to activate NLRP3 for recruiting and interacting with caspase1 precursor (pro-caspase1) through the adaptor molecule ASC (17). Pro-caspase1 is cleaved into active caspase1, which in turn promotes the secretion of mature IL-18 and IL-1β and triggers inflammatory responses.

Adiponectin is an adipose-derived adipokine (18). Much attention has been attracted by adiponectin because of its insulin-sensitizing (19), anti-inflammatory and hepatoprotective properties (20, 21). Previous studies have demonstrated that the plasma level of adiponectin is negatively correlated with NAFLD (18), and hypoadiponectinemia is independently associated with hepatic steatosis and inflammation in NASH patients (22). Adiponectin deficiency can accelerate the progression of steatohepatitis in NASH mouse model and induce severe liver fibrosis (20, 23). However, the potential mechanism underlying the hepatoprotective effect of adiponectin has not been has yet to be fully elucidated. Therefore, this research aimed to explore the effects of adiponectin on palmitic acid (PA)-mediated NLRP3 inflammasome activation in hepatocytes and its potential molecular mechanisms.

## Materials and Methods

### Experimental Animal Model

The ethical approval for this study was obtained from the Animal Ethics Committee of our institution. All animals were maintained and used in accordance with the guidelines and policies approved by the Animal Care and Use Committee of Shanghai Jiao Tong University School of Medicine. To induce the animal model of non-alcoholic steatohepatitis, 4-week-old male C57BL/6 and adiponectin-knockout (adiponectin-KO) mice were randomly assigned to two groups: normal diet feeding group and high fat-diet (HFD) feeding (D12492, Research Diets) group. All animals were given unlimited access to water and food, and kept under a controlled temperature of 22–24°C with a 12-h light/12-h dark cycle. The mice were then euthanized, and their liver tissue and plasma samples were collected at 0, 4, 8, and 12 weeks for further analyses.

### Cell Isolation and Treatment

Hepatocytes were isolated from male C57BL/6 mice by a two-step (collagenase B and pronase E) perfusion method under ketamine/xylazine anesthesia as described previously (24). The isolated hepatocytes were seeded in collagen-coated culture dishes at a density of 2^*^10∧5 cells/ml and cultured in Dulbecco's modified Eagle's medium (HyClone, Logan, UT, USA) supplemented with 10% fetal bovine serum (Gibco, Carlsbad, CA, USA) and 1% (v/v) penicillin-streptomycin at 37°C in 5% CO2 incubator for 48 h. The hepatocytes were then serum-starved for 6 h, followed by treatment with adiponectin (APN; 10 μg/ml, Biovendor, Nycodenz) for 2 h prior to 300 μmol/ml palmitic acid (PA; Sigma, St. Louis, MO) exposure for 24 h. Palmitic acid was conjugated to 2% BSA and dissolved in DMEM, 2%BSA was added as control in untreated group. For the inhibition experiment, the hepatocytes were treated with AMPK inhibitor (compound C, 10 μm/M; Sigma) 2 h prior to adiponectin treatment. The treated hepatocytes and culture supernatant were collected for subsequent analyses.

### Real Time PCR Analysis

Total RNA was extracted from the cultured hepatocytes or liver tissue by using TRIzol reagent (Invitorgen, Carlsbad, California, USA) based on the manufacturer's protocols. cDNA synthesis was performed using SuperScriptIII reverse transcriptase, random primers and 1 μg RNA (Invitrogen, Carlsbad, CA, USA). qPCR was conducted using Power SYBR Green PCR Master Mix (Applied Biosystems, Foster City, CA, USA). The expression level of each gene was normalized to the corresponding housekeeping gene β actin value, and presented as fold changes relative to controls. All primers sequences are shown in Table 1.

**Table 1:** Sequences of primers for quantitative real-time PCR.

	**Gene name**	**Forward primer sequence (5′-3′)**	**Reverse primer sequence (5′-3′)**
Mouse	NLRP3	AAGGCTTGTGTGGGACCAA	GCGCTTCTAAGGCACGTTTT
	IL1β	TGCCACCTTTTGACAGTGATG	TGCCACCTTTTGACAGTGATG
	IL18	ACGTGTTCCAGGACACAACA	ACAGGCGAGGTCATCACAAG
	Caspase1	CGCGGTTGAATCCTTTTCAGAC	CCTTTCCAACAGGGCGTGAA
	ASC	TCCACAGACCCAAGTTATGGC	GGTGCCTTTCTAAGCCCCAT
	TNFa	GATCGGTCCCCAAAGGGATG	ACAAGGTACAACCCATCGGC
	IL6	GGGACTGATGCTGGTGACAA	TCTGCAAGTGCATCATCGTT
	β-actin	TTCGTTGCCGGTCCACACCC	GCTTTGCACATGCCGGAGCC

### Western Blotting

Equivalent amounts of total protein extracted from the hepatocytes or liver tissue were loaded onto 10% sodium dodecyl sulfate poly-acrylamide gel, and subsequently transferred onto polyvinylidene fluoride membranes (Millipore, Bedford, MA, USA). After blocking with 5% non-fat milk, the membranes were incubated overnight with primary antibodies at 4°C, followed by washing with 0.05% Tween-20/TBS. Following incubation with secondary antibodies, the resulting blots were visualized by an enhanced chemiluminescence kit (Pierce Perbio, Rochford, IL) based on the manufacturer's instructions. Finally, the protein bands were digitally scanned, and quantitated using ImageJ software. The antibodies were used for western blotting as folowing: NLRP3 (1:1000), AMPK (1:1000), p-AMPK (1:800), NFκBp65 (1:500) and NFκB p-p65 (1:500) antibodies were purchased from Boaosen, Inc, Beijing, China. Caspase-1 (1:1000), c-Jun terminal kinase (JNK; s1:1000), p-JNK (1:1000), extracellular signal-regulated kinase1/2 (Erk1/2; 1:1000) and p-Erk1/2 (1:1000) antibodies were obtained from Santa Cruz Biotechnology, Inc. IL1β antibody (1:1000) were supplied by Cell Signaling Technology, Inc.

### Immunofluorescence Staining

The expression levels of NLRP3 in hepatocytes exposure to PA were analyzed by immunofluorescence. All images were obtained using a fluorescence microscope (IX51, Olympus, Japan).

### Determination of Reactive Oxygen Species

The level of reactive oxygen species (ROS) in hepatocytes was measured using 2',7'-dichlorofluorescein diacetate (DCFH-DA, ROS probe; D6883, Sigma). All images were acquired using a fluorescence microscope (IX51, Olympus, Japan). The excitation and emission wavelengths were fixed at 488 and 525 nm, respectively.

### Detection of Lipid Droplet Deposition

Hepatocytes were treated as described above and then fixed with 10% formalin. Lipid droplet deposition in cells was detected with BODIPY 493/503 (790389, Sigma), and then observed using a fluorescence microscope (IX51, Olympus, Japan).

### Biochemical Analysis and Cytokine Assay

The levels of aspartate aminotransferase (AST), alanine aminotransferase (ALT), cholesterol (CHOL), and total triglyceride (TG) were detected with the commercial kits (Nanjing Jiancheng Bioengineering Institute). Cytokines in the supernatant or liver tissue were assessed using the commercially available enzyme-linked immunoassay (ELISA) kits. The ELISA kits for IL1β, IL18, TNFα, and IL-6 were obtained from Elabscience (Wuhan, China). In briefly, cell supernatant harvested was centrifugated for 20 min at 1,000 g centrifugation to remove impurities and cell debris. For tissue homogenate preparation, 1 g liver tissue specimen was homogenized in 9 ml PBS on ice. After centrifugation at 10,000 g for 5 min at 4°C, the liquid supernatant was collected for further study. Hundred microliter supernatant or standards were added into per well, and covered the wells and incubated 90 min at room temperature. Then, the solution was discarded, and 100 μl of detection antibody was added to each well and incubated for 1 h at room temperature. After 3 times washing with PBS, 100 μl of working dilution of substrate reagent was added to each well and incubated for 30 min at room temperature. After the last washing, 50 μl of stop solution was added to each well and read at 450 nm immediately. Interassay and intraassay variations <10% were used in this assay.

### Histological Analysis of Liver Tissue

The liver tissue sections were subjected to hematoxylin and eosin (H&E) staining, oil red O (ORO) staining, and immunohistochemistry dye by following the standard protocols as previously described. Briefly, the paraffin-embedded liver sections were deparaffinized, rehydrated. Stained with H&E or ORO, and then observed under a microscope. For immunohistochemical staining, the liver sections were incubated with freshly prepared 3% hydrogen peroxide for 20 min. After washing with PBS, antigen retrieval was conducted in 0.01 M citric acid. The sections were submerged in 5% normal blocking serum for 30 min, and subsequently incubated with NLRP3, caspase1 or IL1β overnight at 4°C. Following that, the sections were incubated with the corresponding secondary antibody for 1 h at room temperature. Lastly, all sections were examined using the microscope.

### Statistical Analysis

All data were presented as mean ± SEM. Student's *t-*test or one-way analysis of variance was employed to compare the statistical differences using SPSS statistics software (version 12.0 for Windows; SPSS, Inc., Chicago, IL, USA). A *p*-value of < 0.05 was deemed as statistically significant. All experiments were repeated at least 3 times.

## Results

### Adiponectin Deficiency Aggravates Liver Injury and Steatosis as Well as Sensitizes to HFD-Induced NLRP3 Inflammasome Activation

**Figure 1:**
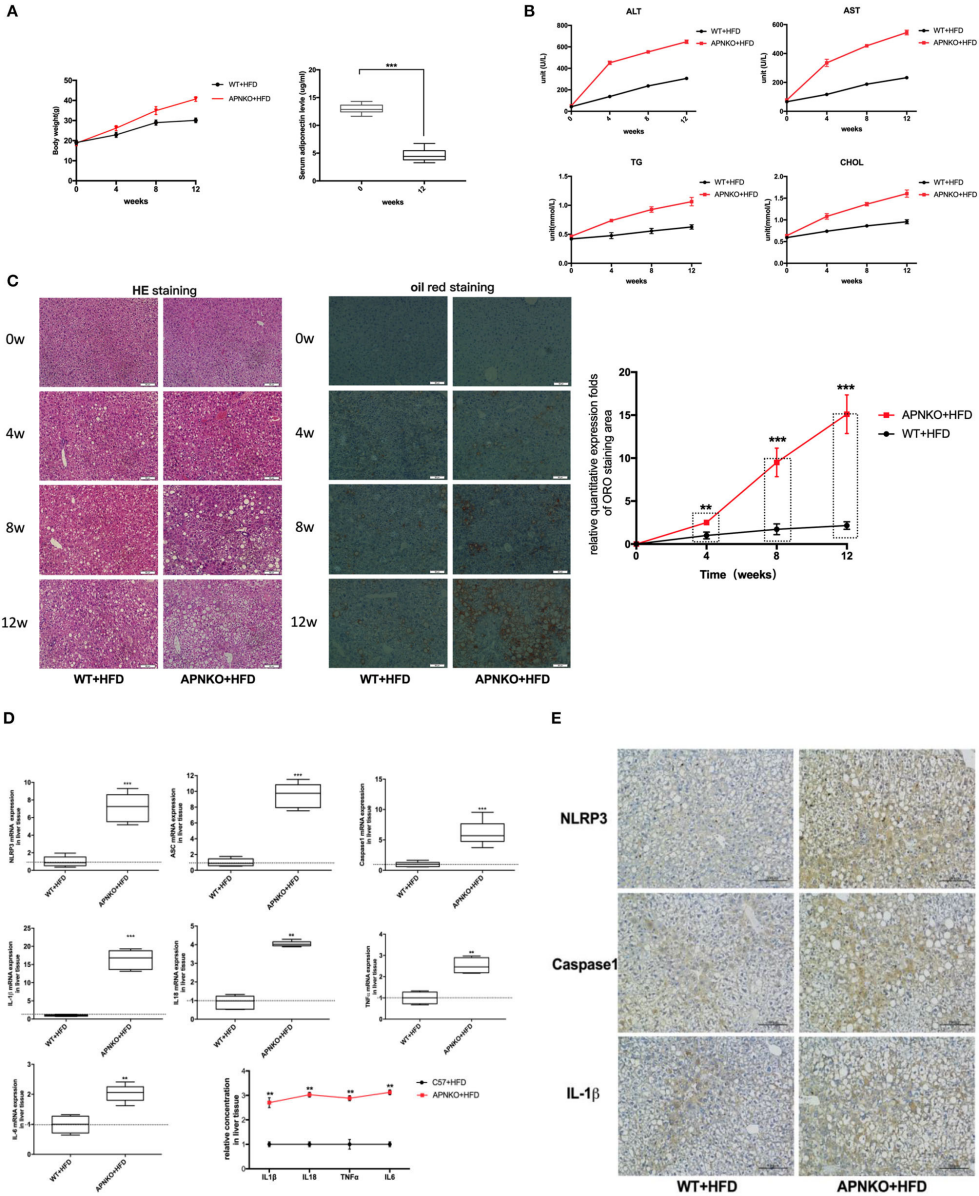
Adiponectin deficiency aggravates liver injury and steatosis as well as sensitizes to HFD-induced NLRP3 inflammasome activation. Four-week-old male wild type (WT) and adiponectin-knockout (APN KO) mice were fed either ND or HFD for 12 weeks. Liver tissue and serum samples were harvested at baseline, 4w, 8w, and 12w, respectively. **(A)** The body weight and the levels of adiponectin after HFD **(B)** the serum levels of ALT, AST, and the concentrations of TG and CHOL were determined by colorimetric assay at baseline, 4w, 8w, and 12w. **(C)** H&E staining and oil red staining in liver tissue sections for evaluation of inflammation and steatosis at different stages, the semi-quantitative analysis of ORO staining was performed by image J. **(D)** NLRP3, ASC, Capase1, IL1β, IL18, TNFα, and IL6 mRNA expression in liver tissue were detected by qPCR. **(E)** NLRP3, caspase1, and IL1β immunohistochemical staining in liver tissue sections from mice-fed with HFD at 12w. Results was presented as mean ± SEM, *n* = 6 mice per group. Differences between two groups were compared using a Student's *t-*tests. Differences between multiple groups were compared using one-way analysis of variance. ^***^p < 0.001, ^**^p < 0.01.

As shown in Figure 1A, high fat diet intake significantly increased body weight in both wildtype and adiponectin deficiency mice, in particular, this change is more significantly adiponectin-KO (APNKO) mice. As expected, high fat diet intake significantly decreased the levels of adiponectin in wildtype mice comparing with that at baseline. To figure out the effects of high-fat-diet (HFD) feeding on hepatic injury and steatosis in APNKO mice, several biochemical markers, and liver histology were analyzed. The results indicated that the serum levels of AST, ALT, CHOL, and TG were markedly increased with the prolongation of HFD feeding time, and appeared to be higher in APNKO mice than in wild-type mice (Figure 1B). H&E and ORO staining revealed severe inflammation, ballooned hepatocytes and worse hepatic steatosis in HFD mice in a time-dependent manner. Moreover, liver injury and fat deposition were more obvious in APNKO mice than in wild-type mice (Figure 1C). To investigate whether NLRP3 inflammasome is involved in HFD-induced liver injury and steatosis under the condition of adiponectin deficiency, the expression levels of NLRP3 inflammasome and related inflammatory markers were detected. As shown in Figure 1C, adiponectin deficiency promoted the gene expression of NLRP3, ASC, caspase1 and IL1β, IL18, TNFα, and IL6 in HFD-fed APNKO mice compared to wild-type mice. Additionally, the concentration of IL1β, IL18, TNFα, and IL6 in liver tissue lysate were remarkably higher in adiponectin-KO mice than in wild-type mice (Figure 1D). In accordance to the above findings, immunohistochemical staining of the liver tissue sections further confirmed that NLRP3, caspase1, and IL1β expression were remarkably upregulated in HFD-fed adiponectin-KO mice compared to wild-type mice (Figure 1E). These data strongly indicate that adiponectin deficiency aggravates HFD-induced liver injury and steatosis as well as sensitizes to HFD-induced NLRP3 inflammasome activation and downstream cytokine production.

### The Effects of Adiponectin Deficiency on ROS Production and NFκB/AMPK/MAPK Signaling Pathways

**Figure 2:**
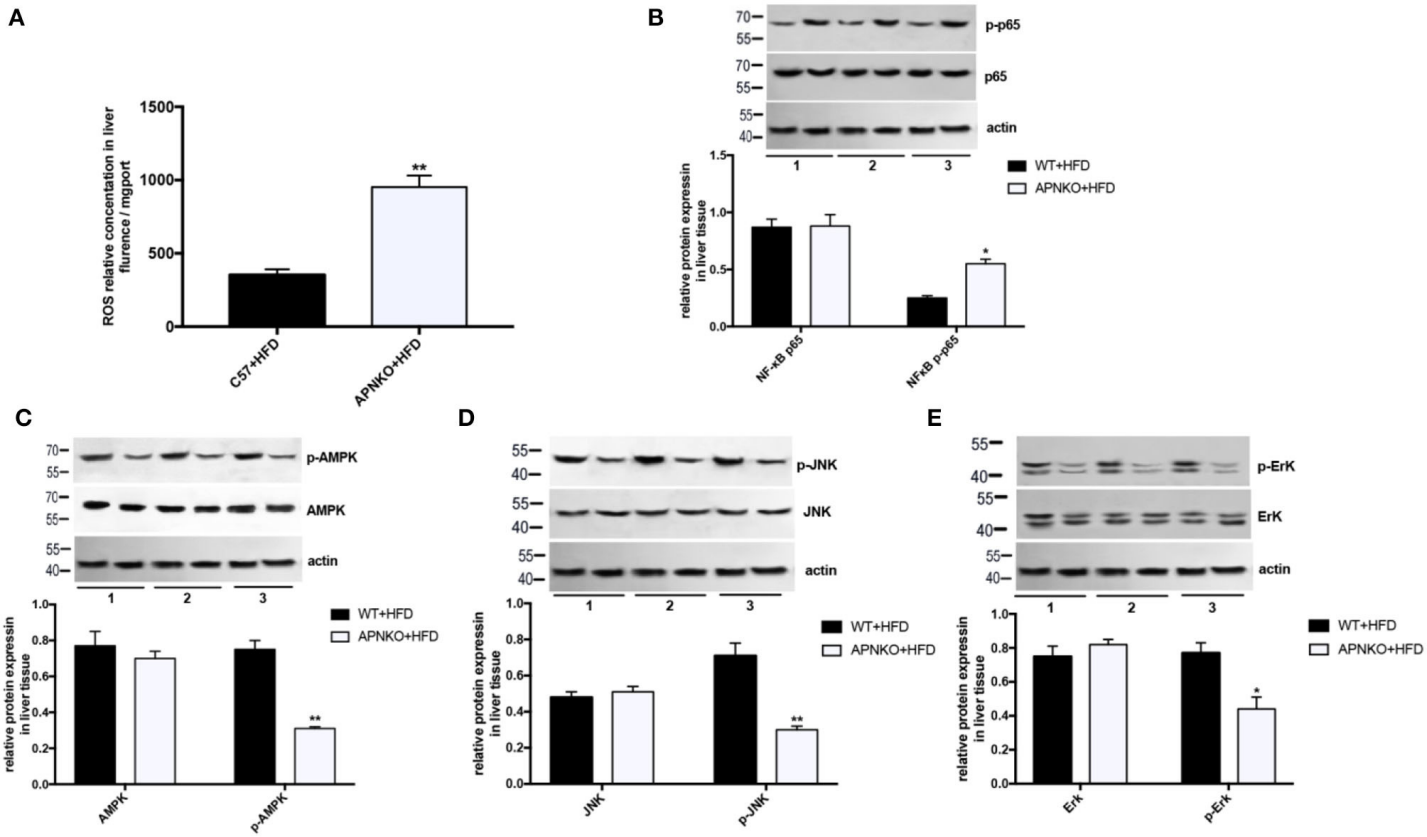
The effects of adiponectin deficiency on ROS production and NFκB/AMPK/MAPK signaling pathways. **(A)** The ROS levels in liver tissue **(B)** NFκB and **(C–E)** AMPK/ p-AMPK, JNK/ p-JNK and ErK1/2/p-ErK1/2 protein expression was detected by quantitative analysis of WB, quantitative data were normalized by actin. The same image for actin has been reused in this figure because the loading control and involved protein was from the same batch of samples, in the case of loading equivalent amounts of total protein, actin were only detected once on a separated membrane to observe whether the internal parameter bands are consistent among the samples. Results was presented as mean ± SEM, *n* = 6 mice per group, Differences between two groups were compared using a Student's *t-*tests. Differences between multiple groups were compared using one-way analysis of variance ^**^p < 0.01, ^*^p < 0.05.

To further elucidate the possible regulatory mechanism by which adiponectin deficiency aggravates HFD-induced NLRP3 inflammasome activation, the levels of ROS production as well as NFκB, AMPK, and MAPK (JNK and ErK1/2) signaling pathways were determined. As presented in Figure 2A, ROS production was significantly elevated in the liver of HFD-fed adiponectin-KO mice compared to wild-type mice. Besides, NFκB expression was markedly upregulated in HFD-fed adiponectin-KO mice compared to wild-type mice (Figure 2B). On the contrary, the phosphorylation levels of AMPK, JNK, and ErK1/2 were markedly reduced in adiponectin deficiency mice, while there was no remarkable alteration in the protein levels of total AMPK, JNK, and ErK1/2 (Figures 2C–E). These results suggest that adiponectin deficiency promotes HFD-induced NLRP3 inflammasome activation pathways (NFκB and ROS) and attenuates AMPK/JNK/ErK1/2 signaling pathways.

### Adiponectin Alleviates PA-Mediated NLRP3 Inflammasome Expression in Hepatocytes

**Figure 3:**
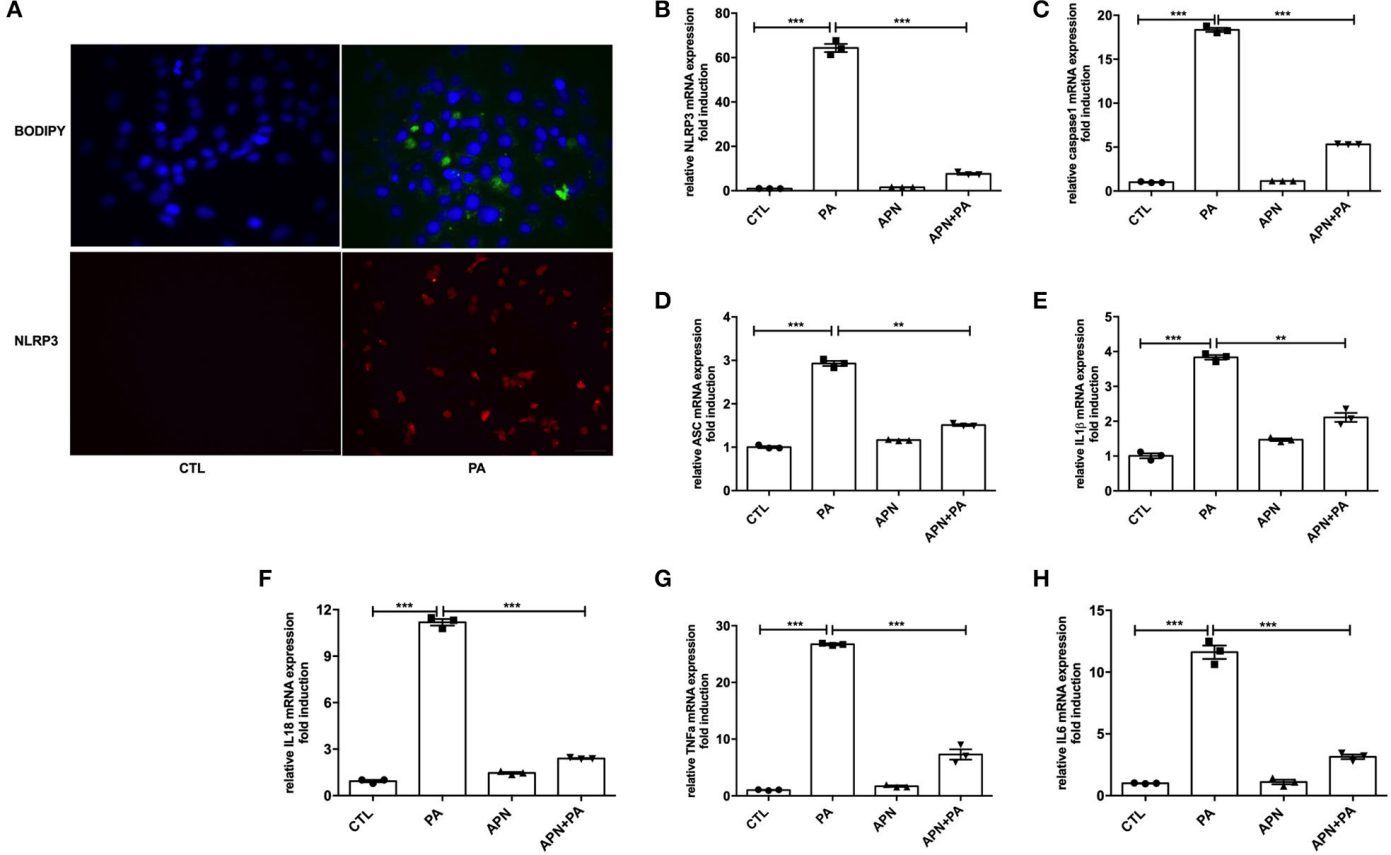
Adiponectin alleviates PA-mediated NLRP3 inflammasome expression in hepatocytes. Hepatocytes were isolated from mice and cultured for 48 h, then cells were serum-starved for 6 h and exposed to palmitic acid (PA, 300 μmol/ml) for 24 h. For inhibition experiment, adiponectin (APN, 10 μg/ml) was used 2 h prior to palmitic acid treatment. **(A)** Lipid droplet deposition assay was determined using BODIY fluorescence dye; the expression of NLRP3 was detected by immunofluorescence. **(B–H)** The expression levels of NLRP3 inflammasome complex genes such as NLRP3/ASC/Caspase1/IL1β/IL18 and other inflammatory makers (TNFα and IL6) were determined by qPCR. Results are presented as means ± SEM, all experiments were performed at least three times and at least in triplicate. Differences between two groups were compared using a Student's *t-*tests. Differences between multiple groups were compared using one-way analysis of variance. ^***^p < 0.001, ^**^p < 0.01.

Taking into account that adiponectin deficiency aggravates HFD-mediated NLRP3 inflammasome expression, we further determine the effects of PA and adiponectin on NLRP3 inflammasome activation in hepatocytes. As presented in Figure 3A, treatment with PA induced lipid droplet deposition and NLRP3 inflammasome expression in hepatocytes. To verify the roles of adiponectin in PA-mediated hepatic activation of NLRP3 inflammasome complexes, hepatocytes were pre-treated with adiponectin for 2 h and then exposed to PA for 24 h. It was found that PA induced the expression levels of NLRP3, caspase1, ASC, IL1β, IL18, and other inflammatory markers (e.g., TNFα and IL6) when compared to control group. However, treatment with adiponectin could alleviate PA-mediated NLRP3 inflammasome activation (Figures 3B–H). These findings reveal that adiponectin exerts a protective effect on PA-stimulated NLRP3 inflammasome activation in hepatocytes.

### AMPK Inhibitor Attenuates Adiponectin-Mediated Hepatoprotective Effects Against PA-Mediated NLRP3 Inflammasome Activation

**Figure 4:**
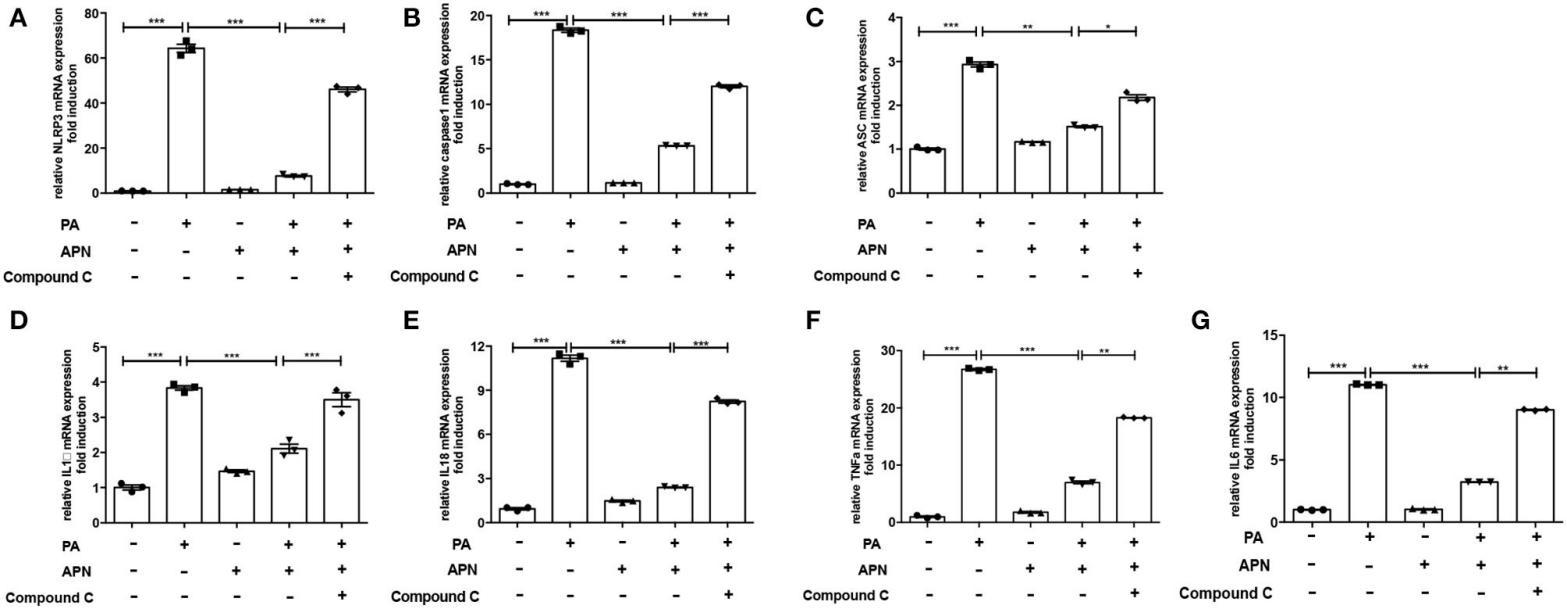
AMPK inhibitor reverses the inhibitory role of adiponectin on PA-induced NLRP3 inflammasome expression. Hepatocytes were pre-treated with APN and/or Compound C for 2 h prior to incubation with PA for 24 h. **(A)** NLRP3, **(B)** caspase1, **(C)** ASC, **(D)** IL-1β, **(E)** IL-18, **(F)** TNFα and (G) IL6 mRNA expression were detected by qPCR. Results are presented as means ± SEM, all experiments were performed at least three times and at least in triplicate. Differences between two groups were compared using a Student's *t-*tests. Differences between multiple groups were compared using one-way analysis of variance. ^***^p < 0.001, ^**^p < 0.01, ^*^p < 0.05.

**Figure 5:**
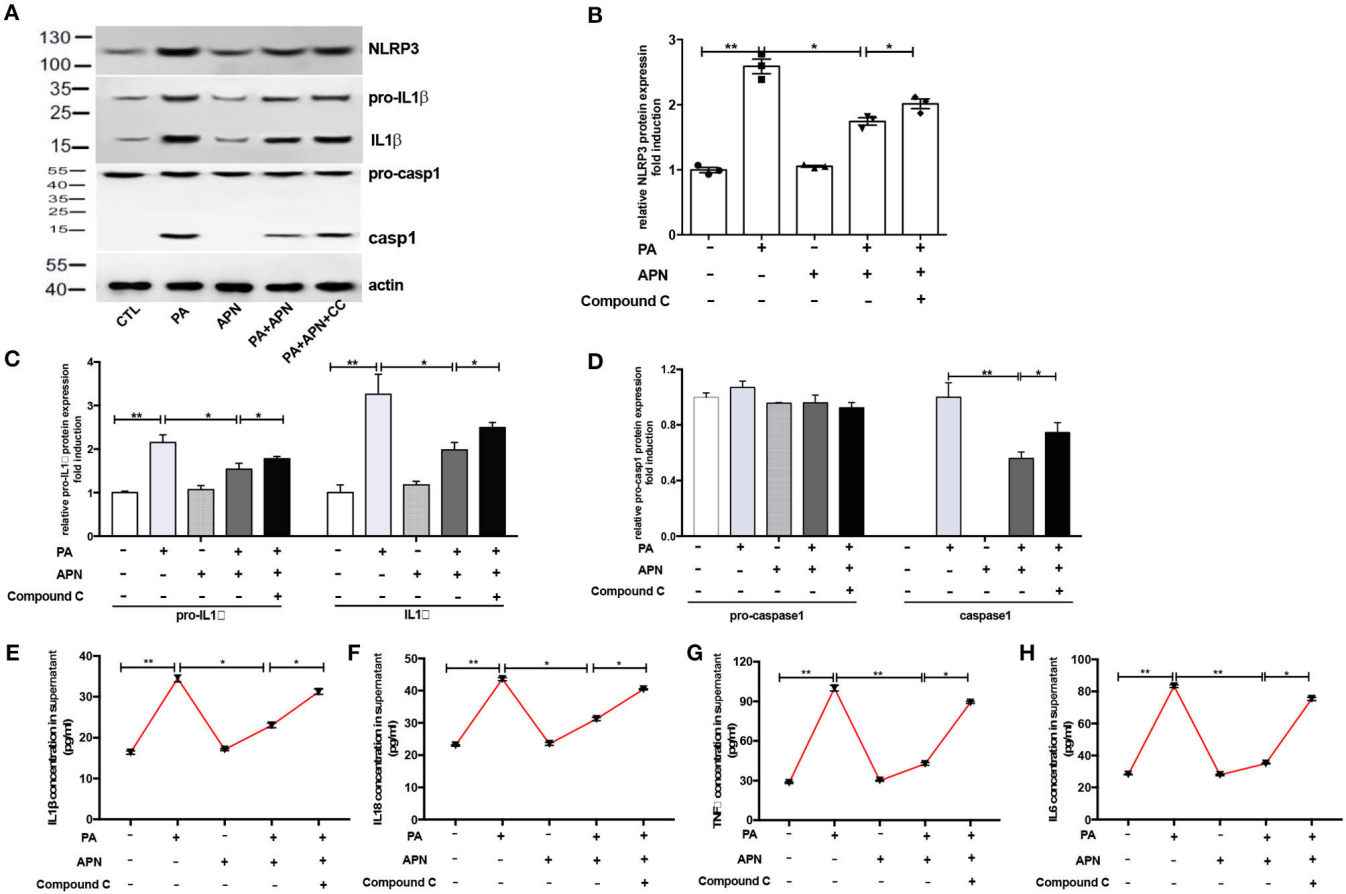
AMPK inhibitor attenuates adiponectin-mediated hepatoprotective effects against PA-mediated NLRP3 inflammasome activation. Hepatocytes were pre-treated with APN 2 h prior to incubation with PA for 24 h. For AMPK inhibition experiment, Compound C (CC, 10 μM) were using 2 h prior to adiponectin. Cell and culture supernatant were harvested for further experiment **(A)** Western blot images and quantified expression of NLRP3 **(B)**, Procaspase1, Caspase1 **(C)**, pro-IL1β and IL1β **(D)**. Quantitative data were normalized by actin. **(E–H)** The concentration of inflammatory cytokines IL1β, IL18, TNFα, and IL6 in cell culture supernatant were measured by ELISA. Results was presented as mean ± SEM, all experiments were performed at least three times and at least in triplicate. Differences between two groups were compared using a Student's *t-*tests. Differences between multiple groups were compared using one-way analysis of variance. ^**^p < 0.01, ^*^p < 0.05.

A previous study has suggested that AMPK can play a vital role in the biological activities of adiponectin (25). The results of *in vivo* experiments showed that the level of p-AMPK was reduced in adiponectin-KO mice fed a HFD. Thus, in the *in vitro* study, the effects of AMPK signaling pathway on NLRP3 inflammasome suppression by adiponectin were assessed with Compound C (10 μm/ml, an AMPK inhibitor). It was found that the mRNA expression levels of NLRP3 (Figure 4A), Caspase1 (Figure 4B), ASC (Figure 4C), IL1β (Figure 4D), IL18 (Figure 4E) TNFα (Figure 4F), and IL6 (Figure 4G) were markedly upregulated in Compound C+adiponectin+PA group compared to adiponectin+PA group (Figure 4), suggesting that compound C can disrupt the protective role of adiponectin against PA-mediated NLRP3 inflammasome activation. To further confirm these findings, the protein expression levels of NLPR3, pro-capase1, caspase1 (P10), pro-IL1β, and IL1β in hepatocytes were detected. Consistent with the above data, adiponectin abolished PA-mediated expression of NLRP3 inflammasome protein complexes. On the contrary, Compound C reversed the inhibitory effect of adiponectin on PA-mediated NLRP3 inflammasome activation in hepatocytes (Figures 5A–D), In support of the above-mentioned data, similar results were also observed with regard to the concentrations of IL1β, IL18, TNFα, and IL6 in cell culture supernatant (Figures 5E–H).

### Adiponectin Plays a Protective Role Against PA-Mediated NLRP3 Inflammasome Activation via AMPK-JNK/Erk-NFkB/ROS Signaling Pathways

**Figure 6:**
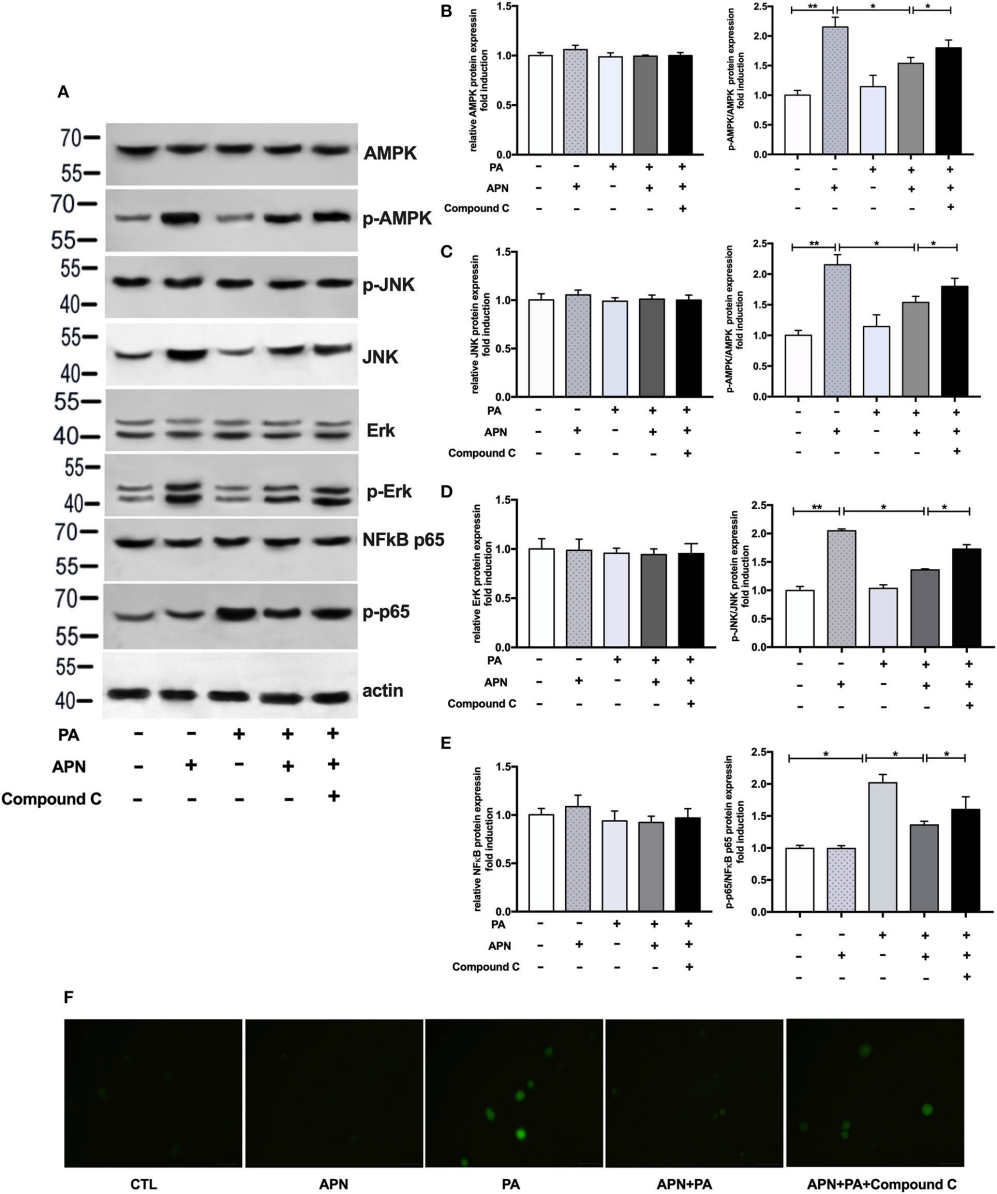
Adiponectin plays a protective role against PA-mediated NLRP3 inflammasome activation via AMPK-JNK/Erk1/2-NFκB/ROS signaling pathways. Hepatocytes were pretreated with APN 2 h prior to incubation with PA for 24 h. For AMPK inhibition experiment, Compound C (10 μM) were using 2 h prior to adiponectin. **(A)** Western blot images and quantified levels of **(B–E)** total AMPK and phosphorylated AMPK, total JNK, and phosphorylated-JNK, total ErK1/2 and phosphorylated-ErK1/2, total NFκB p65, and phosphorylated-p65. Quantitative data were normalized by actin. **(F)** The ROS production in hepatocytes was detected by BODIPY fluorescence dye. Results was presented as mean ± SEM, all experiments were performed at least three times and at least in triplicate. Differences between two groups were compared using a Student's *t-*tests. Differences between multiple groups were compared using one-way analysis of variance. ^**^p < 0.01, ^*^p < 0.05.

Based on the above data, we speculated that adiponectin may inhibit PA-mediated inflammasome activation through AMPK signaling pathway. Therefore, we next determined the expression levels of AMPK and other related signaling pathways. As presented in Figure 6A, adiponectin increased the phosphorylation levels of AMPK, JNK, and ErK1/2 in hepatocytes. Our previous work has indicated that AMPK can act as an upstream regulator of JNK and Erk1/2 in hepatic stellate cells (26). Thus, we also detected the activities of JNK and Erk1/2 following the exposure to AMPK inhibitor. Our results demonstrated that Compound C markedly decreased adiponectin-induced expression levels of p-AMPK, p-JNK, and p-Erk1/2 (Figures 6A–D), suggesting that AMPK can regulate p-JNK and p-Erk1/2 as upstream signaling pathways. Given that PA could induce NFκB phosphorylation and ROS production (Figures 6A–F) that act as the regulators of NLRP3 inflammasome activation (12), we further assessed the effects of adiponectin and Compound C on PA-mediated NFκB and ROS expression, It was found that adiponectin inhibited PA-mediated NFκB phosphorylation and ROS production in hepatocytes, and Compound C reversed the suppressive effects of adiponectin on PA-mediated NFκB expression and ROS production (Figure 6F). These findings reveal that adiponectin can suppress PA-mediated NLRP3 inflammasome activation in hepatocytes via AMPK-JNK/Erk1/2-NFκB/ROS signaling pathways.

## Discussion

Accumulating evidence has suggested that adiponectin exerts beneficial effects on hepatic disorders (20, 23), including steatohepatitis and liver fibrosis (26, 27). However, the potential mechanism responsible for these effects has been not yet fully elucidated. Recently, several studies have shown that adiponectin and adiponectin receptor agonist (adipoRon) exhibit protective effects against diabetes-related vascular disorders or nephropathy by suppressing NLRP3 activation (28, 29). However, it remains unclear whether adiponectin can affect NLRP3 inflammasome expression in hepatic diseases and its potential underlying mechanism. Therefore, in this study, we explored the effect and potential mechanism of adiponectin on PA-mediated NLRP3 inflammasome activation through both *in vitro* and *in vivo* experiments. The *in vivo* findings revealed that adiponectin deficiency aggravated HFD-induced liver injury and steatosis as well as stimulated NLRP3 inflammasome activation. Meanwhile, in *in vitro* study, adiponectin treatment suppressed PA-mediated NLRP3 inflammasome activation and ROS production in hepatocytes. To our knowledge, this work for the first time demonstrated that the protective effect of adiponectin against PA-mediated NLRP3 inflammasome activation in hepatocytes was attributed to AMPK-JNK/ErK1/2-NFκB/ROS signaling pathways.

Adiponectin plays important roles in the metabolic functions of adipose tissue, skeletal muscle and liver (30). Previous studies have shown that the serum levels of adiponectin are reduced in NASH patients (31), and hypoadiponectinemia is negatively associated with steatosis and inflammation in NAFLD patients (32). Adiponectin-deficient mice exist hepatic excessive steatosis and necroinflammation in a mouse model of non-alcoholic steatohepatitis (20, 23). Consistent with these findings, our *in vivo* study also demonstrated that adiponectin deficiency aggravated HFD-induced liver injury, such as elevated serum ALT/AST levels, promoted lipid droplet accumulation and increased ROS production. In addition, it was found that adiponectin deficiency promoted HFD-induced NLRP3 inflammation activation and NFκB expression, and attenuated the phosphorylation levels of AMPK, JNK, and Erk1/2. Several studies have indicated that NLRP3 inflammasome can be activated by endotoxin, ROS, uric acid, etc. (16, 33), and adiponectin may prevent the progression of steatohepatitis by regulating oxidative stress (23). Therefore, we speculated that the aggravating effect of adiponectin deficiency on NLRP3 inflammasome activation was due to the increased ROS production and activated NFκB signaling pathway. The underlying molecular mechanisms were further explored *in vitro*.

Previous studies have reported that the increased levels of free fatty acids involved in the pathogenesis of NASHs (6), and palmitic acid, as a saturated fatty acid, has been implicated in trigger inflammation and leading to liver injury (34). Also, there are several studies presenting that palmitic acid could induce the expression of NLRP3 inflammasome in liver cells and favor inflammatory responses (7, 10). The results of *in vitro* experiments showed that PA stimulated NLRP3 inflammasome activation in hepatocytes, promoted lipid droplet accumulation, increased ROS production, and activated NF-κB signaling. Interestingly, we observed that adiponectin treatment could abolish PA-mediated NLRP3 inflammasome activation and promote the activation of AMPK, JNK, and ErK1/2 signaling pathways in hepatocytes. In contrast, AMPK inhibitor reversed the inhibitory effects of adiponectin on NLRP3 inflammasome activation, and suppressed the activation of APMK, JNK, and ErK1/2. These data indicate that AMPK activation is necessary for the protection of adiponectin against NLRP3 inflammasome activation. Furthermore, JNK/Erk1/2 are the downstream effectors of adiponectin. It was found that adiponectin decreased PA-mediated ROS generation and attenuated NF-kB signaling, and AMPK inhibitor reversed these effects of adiponectin. Based on our present results and previous findings showing that NLRP3 activation require two signals: the priming signal is mediated by NFκB pathway (16), while the activating signal is associated with ROS generation, K+efflux and the lysosome membrane permeabilization (33, 35, 36), we postulated that AMPK-JNK/Erk/-NFκB/ROS pathways are responsible for the inhibition of PA-mediated NLRP3 inflammasome by adiponectin. Although further research is needed to verify these molecular mechanisms, interestingly, in support of our data, similar signal transduction events (APN-AMPK-ROS pathways) were also involved in the therapeutic effects of adiponectin on monocytic THP-1 cells (37).

## Conclusion

The findings of the current study revealed the effect of adiponectin on NLRP3 inflammasome and its molecular mechanisms, in which adiponectin inhibited PA-mediated NLRP3 inflammasome activation in hepatocytes. Adiponectin analogs or AMPK agonists could serve as a potential novel agent for preventing or delaying the progression of NASH and NAFLD.

## Data Availability Statement

All datasets generated for this study are included in the article/supplementary material.

## Ethics Statement

The animal study was reviewed and approved by the animal ethics committee of Shanghai Jiao Tong University, School of Medicine.

## Author Contributions

ZD and XW designed and performed experiments, analyzed data, and wrote the manuscript. ZD, QZ, MN, XY, SW, and LL contributed to analysis and interpretation of data and assisted in the preparation of the manuscript. ZD and QZ performed experiments. XW supervised the project and contributed to manuscript development. All authors contributed to the article and approved the submitted version.

## Conflict of Interest

The authors declare that the research was conducted in the absence of any commercial or financial relationships that could be construed as a potential conflict of interest.
